# Transfusion Indication Threshold Reduction (TITRe2) randomized controlled trial in cardiac surgery: statistical analysis plan

**DOI:** 10.1186/s13063-015-0564-x

**Published:** 2015-02-22

**Authors:** Katie Pike, Rachel L Nash, Gavin J Murphy, Barnaby C Reeves, Chris A Rogers

**Affiliations:** Clinical Trials and Evaluation Unit, School of Clinical Sciences, University of Bristol, Bristol, UK; Department of Cardiovascular Sciences, University of Leicester, Leicester, UK

**Keywords:** Cardiac surgery, Red cell, Restrictive, Statistical analysis plan, Transfusion

## Abstract

**Background:**

The Transfusion Indication Threshold Reduction (TITRe2) trial is the largest randomized controlled trial to date to compare red blood cell transfusion strategies following cardiac surgery. This update presents the statistical analysis plan, detailing how the study will be analyzed and presented. The statistical analysis plan has been written following recommendations from the International Conference on Harmonisation of Technical Requirements for Registration of Pharmaceuticals for Human Use, prior to database lock and the final analysis of trial data. Outlined analyses are in line with the Consolidated Standards of Reporting Trials (CONSORT).

**Methods and design:**

The study aims to randomize 2000 patients from 17 UK centres. Patients are randomized to either a restrictive (transfuse if haemoglobin concentration <7.5 g/dl) or liberal (transfuse if haemoglobin concentration <9 g/dl) transfusion strategy. The primary outcome is a binary composite outcome of any serious infectious or ischaemic event in the first 3 months following randomization.

The statistical analysis plan details how non-adherence with the intervention, withdrawals from the study, and the study population will be derived and dealt with in the analysis. The planned analyses of the trial primary and secondary outcome measures are described in detail, including approaches taken to deal with multiple testing, model assumptions not being met and missing data. Details of planned subgroup and sensitivity analyses and pre-specified ancillary analyses are given, along with potential issues that have been identified with such analyses and possible approaches to overcome such issues.

**Trial registration:**

ISRCTN70923932.

## Update

This paper provides the detailed statistical analysis plan for the Transfusion Indication Threshold Reduction (TITRe2) randomized controlled trial, comparing transfusion rates, morbidity and healthcare resource use following two different transfusion strategies after cardiac surgery.

## Background

Perioperative anaemia is common after cardiac surgery, and transfusion of allogeneic red blood cells (RBCs) is the preferred treatment for acute anaemia. Observational studies suggest that transfusion is harmful after cardiac surgery [1-3]; by contrast, randomized controlled trials of restrictive (lower haemoglobin level) versus more liberal (higher haemoglobin level) RBC transfusion thresholds have not demonstrated adverse effects attributable to transfusion [4]. Uncertainty about a safe restrictive RBC transfusion threshold in cardiac surgery persists and is reflected in transfusion rates across cardiac centres ranging from 25 to 75% [5] in the UK and 8 to 93% [6] in the USA. The TITRe2 trial has been established to address the current uncertainty around safe haemoglobin levels for RBC transfusion after cardiac surgery.

## Methods and Design

TITRe2 is a multicentre, UK-wide, open parallel group randomized controlled trial. It is the largest randomized controlled trial to date (2,000 randomized patients) to compare RBC transfusion strategies following cardiac surgery. Patients are randomized in a 1:1 ratio to one of two RBC transfusion strategies: (a) a ‘restrictive’ threshold, whereby transfusions are given if the haemoglobin concentration is below 7.5 g/dl (or haematocrit <22%), or (b) a ‘liberal’ threshold, whereby transfusions are given if the haemoglobin concentration is below 9 g/dl (or haematocrit <27%). Cohort minimization is used to minimize imbalance of: (a) centre and (b) operation type. The study population is all adult patients (aged 16 or over) undergoing non-emergency elective cardiac surgery (this includes non-emergency cases admitted from home and non-emergency inpatient cases). Eligibility criteria are as inclusive as possible, to promote the applicability of the evidence obtained during the trial. Full details of the study background and design have been reported elsewhere [7].

Following recommendations from the International Conference on Harmonisation of Technical Requirements for Registration of Pharmaceuticals for Human Use [8], a pre-specified detailed statistical analysis plan has been written prior to database lock and final analysis of the trial data. However, during the course of the study, analysis requested by the Data Monitoring and Safety Committee has been performed prior to finalization of this statistical analysis plan. This analysis comprised monitoring of recruitment rates and data completeness, monitoring of demographic data, and descriptive comparisons of safety data, including the primary outcome measure, by masked treatment allocation. At a pre-planned interim analysis carried out after half the participants had been followed up, a formal comparison was performed for the primary outcome measure only. No formal comparisons were performed at any other time.

### Study objectives

The objectives of the randomized controlled trial are to: (a) estimate the difference in the risk of a postoperative infection or ischaemic event between restrictive and liberal transfusion thresholds; (b) compare the effects of restrictive and liberal transfusion thresholds with respect to a range of secondary outcomes; (c) estimate the cost-effectiveness of the restrictive compared with the liberal haemoglobin transfusion threshold and describe this in terms of a cost-effectiveness acceptability curve. A UK National Health Service Research Ethics Committee (Oxfordshire C) approved the study (08/H0606/125). The trial is registered (ISRCTN70923932).

### Outcomes

The primary outcome measure is a binary composite outcome of any serious infectious (sepsis or wound infection) or ischaemic (permanent stroke, myocardial infarction, acute kidney injury or gut infarction) event in the first 3 months after randomization. Full details of qualifying events and the manner in which they will be verified are available in the protocol [7].

Secondary outcome measures are:

- Units of RBCs and other blood components transfused during a participant’s hospital stay,
- Proportion of patients experiencing an infectious event,
- Proportion of patients experiencing an ischaemic event,
- EQ5D [9],
- Duration of postoperative stay in intensive care or high dependency unit,
- Duration of postoperative hospital stay,
- All-cause mortality,
- Significant pulmonary morbidity, comprising: (a) initiation of non-invasive ventilation (for example, continuous positive airway pressure ventilation), (b) reintubation/ventilation, or (c) tracheostomy,
- Cumulative resource use, cost and cost-effectiveness. (This analysis is being undertaken by the Health Economics Research Centre at the University of Oxford and is not covered in this statistical analysis plan.)

### Sample size

Based on previous data [1] and allowing for anticipated non-adherence to the allocated thresholds [7], the primary outcome frequencies were hypothesized to be 17% and 11% in the liberal and restrictive groups. A sample size of 1,468 was required to detect this difference with 90% power and 5% significance (two-sided test). The target sample size was inflated to 2,000 to allow for uncertainty about non-adherence, since higher than expected non-adherence would reduce power. Full details are reported elsewhere [7].

### Flow of participants

**Figure 1:**
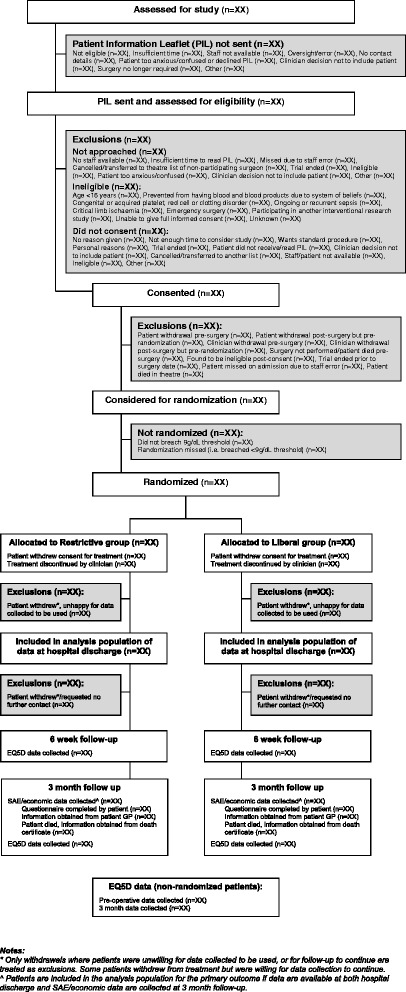
**Flow of participants.**

The flow of participants will be described using a flowchart (see Figure 1) as recommended by the Consolidated Standards of Reporting Trials (CONSORT) [10].

Participants consent to the study before surgery if they meet all of the pre-consent eligibility criteria (see Figure 1) and give written consent. They are then randomized if at any point post-surgery they meet the post-consent eligibility criteria (haemoglobin concentration falls below 9 g/dl or haematocrit below 27%). This means that a significant proportion of patients (estimated to be approximately 30% from earlier studies [1]) consent to the study but are not randomized. Randomized patients should be given a RBC transfusion as soon as possible after haemoglobin or haematocrit levels breach the relevant thresholds, and at most within 24 hours.

### Withdrawals

Patients can withdraw their consent for the study at any time; reasons for withdrawal are collected, along with instructions as to (a) whether data already collected can be used and (b) whether the patient is happy to participate in follow-up sessions. In addition, clinicians can choose to permanently discontinue treatment according to the protocol for a patient; this does not constitute a withdrawal and data collection continues as planned but transfusions need no longer be given according to the study protocol.

### Patient population

The analysis population will consist of all randomized patients, excluding: (a) patients marked as ‘randomized in error’ and (b) withdrawn patients who were unwilling for data collected to be used. Randomization in error is expected to happen rarely (<10 patients); it occurs when a member of research staff realizes shortly after randomization and prior to any intervention that a randomized participant is not in fact eligible. All study analyses will be performed on a modified intention-to-treat basis (including all randomized patients, with the exception of withdrawn patients or those with missing outcome data).

### Adherence to the study protocol

Assumptions regarding transfusion rates in the two groups were made in calculating the sample size [7]; if transfusion rates do not match these assumptions, the power of the study will be reduced. Therefore, measuring and assessing adherence with the transfusion protocol is critical. Non-adherence is defined in two ways: (a) the patient received a RBC transfusion outside of the protocol (‘extra’ transfusion) and (b) the patient was not given a RBC transfusion that, according to the protocol, should have been given (‘withheld’ transfusion). Adherence will be assessed for the period from randomization to hospital discharge, although if a patient withdraws or has treatment discontinued, adherence after the time of withdrawal or discontinuation will not be assessed. For both types of non-adherence, instances will be classified as mild, moderate or severe (see Table 1), according to the likely influence on transfusion rates, and therefore possible influence on study outcomes.

**Table 1:** **Non-adherence to transfusion protocol**

	**Transfusion outside of protocol**	**Transfusion according to protocol withheld**
Mild	Not applicable	A transfusion took place, but more than 24 hours after the breach of the relevant transfusion threshold
Moderate	Patient transfused, but patient did breach the relevant threshold for transfusion at some point postoperatively (before or after the transfusion outside of protocol)	Patient was not transfused following a breach, but the patient had previously had at least one post-randomization transfusion
Severe	Patient transfused, and patient did not breach the relevant threshold for transfusion at any point postoperatively	Patient was not transfused following a breach, and patient had no post-randomization transfusions

A patient can breach the relevant threshold for transfusion several times, and so there can be more than one case of non-adherence per patient.

The frequency of each type of non-adherence will be described by treatment allocation. Further descriptive analyses will be undertaken, to look at non-adherence in more detail, including: reasons for non-adherence, number of deviations from the protocol per patient, haemoglobin or haematocrit levels at deviations and the day of the week and time of the day of deviations. Characteristics of patients with or without any non-adherence will be compared and non-adherence rates will be described by site.

### Statistical analysis principles

Analysis principles and presentation of data will follow the guidance issued in the CONSORT statement [10].

### Descriptive data

Pre-randomization characteristics (for example, patient demography, intra-operative details and pre-randomization RBC transfusions) will be described by treatment allocation for patients in the analysis population. Continuous variables will be summarized using the mean and standard deviation (or median and interquartile range if the distribution is skewed), and categorical data will be summarized as a number and percentage. Any imbalances in the characteristics of the patients will be described but statistical tests for imbalance will not be carried out in line with recommendations [10]. In addition, available characteristics will be described by: (a) non-consented and consented patients and (b) consented but not randomized (because threshold was not breached) and randomized patients (including pre- and intra-operative characteristics, transfusions, haemoglobin levels, EQ5D data and mortality).

### Outcome data

All outcomes listed in the study protocol will be analyzed under the umbrella of one of four types of outcome: (a) binary, (b) continuous, (c) time to event and (d) continuous longitudinal. Table 2 classifies each outcome.

**Table 2:** **Classification of primary and secondary outcomes**

**Category**	**Outcomes**
Binary outcome measures	• Primary outcome measure: proportion of patients experiencing an infectious or ischaemic event
	The following secondary outcome measures:
	• Proportion of patients experiencing an infectious event
	• Proportion of patients experiencing an ischaemic event
	• Use of activated factor seven
	• Use of Human Blood Coagulation Factor IX
	• Significant pulmonary morbidity
Continuous outcome measures	The following secondary outcome measures:
	• Units of RBCs transfused
	• Fresh frozen plasma transfusions
	• Cryoprecipitate transfusions
	• Platelet transfusions
Time-to-event outcome measures	The following secondary outcome measures:
	• Time from randomization to first occurrence of the primary outcome measure (secondary analysis of the primary outcome measure)
	• Duration of post-randomization stay in intensive care or high dependency unit
	• Duration of post-randomization hospital stay
	• Time from randomization to death from any cause
Continuous longitudinal outcome measures	The following secondary outcome measures:
	• EQ5D single summary index score
	• EQ5D visual analogue scale score

General presentation and analysis techniques for each type of outcome are described next.

#### Binary outcomes

The numbers and percentages of patients experiencing each outcome will be presented by treatment group and compared using logistic regression. Formal statistical comparisons of treatment effects will only be performed if more than ten patients in total experience the outcome (with at least one event in each treatment group). Treatment comparison estimates will be presented as adjusted odds ratios and 95% confidence intervals.

#### Continuous outcomes

These will be summarized by the mean and standard deviation (or median and interquartile range if data are skewed) in each treatment group and compared using linear regression. For untransformed data, treatment comparisons will be presented as adjusted differences in means with 95% confidence intervals, and for logarithmically transformed data as adjusted ratios of geometric means with 95% confidence intervals. If a logarithmic transformation is not satisfactory other analysis or presentation methods will be sought.

#### Time-to-event outcomes

These will be summarized by the median and interquartile range in each treatment group and compared using Cox’s proportional hazards models, with treatment comparisons presented as hazard ratios and 95% confidence intervals. Such models require an assumption of proportional hazards to be met. Any patients with a time of zero (for example, the duration of post-randomization stay in an intensive care or high dependency unit might be zero if the patient was randomized after being discharged from the intensive care or high dependency unit) will be included in analyses by assuming a time of half of the smallest non-zero time to the event. Appropriate censoring variables will be used, as given in Table 3.

**Table 3:** **Censor variables for time-to-event outcomes**

**Outcome**	**Censor variable**
Time from randomization to first occurrence of primary outcome	Date of 3 month follow-up questionnaire, if completed
	Date of death, for patients who die prior to 3 month follow-up
	Date of discharge from hospital, for patients who survive 3 months postoperatively but do not complete the follow-up questionnaire (which captures primary outcome events after hospital discharge)
Duration of post-randomization stay in intensive care or high dependency unit	Time of death in intensive care or high dependency unit
Duration of postoperative hospital stay	Time of death in hospital
Time to death	Time of last follow-up (usually 3 months post-operation)

#### Continuous longitudinal outcomes

These will be compared using a linear mixed-effects methodology with the treatment group and study design variables fitted as fixed effects, and patient terms as random effects. Separate parameter estimates will be incorporated into models for: (a) the mean baseline response across both treatment groups and (b) each post-randomization time point for each treatment. This approach of ‘jointly’ modelling the baseline and post-intervention measurements avoids the necessity of either excluding cases with missing baseline measures or imputing missing baseline values. If the time by treatment interaction (post-intervention) is not statistically significant at the 10% level, an overall treatment effect will be reported. If the interaction is statistically significant, the changes in treatment effect with time will be described. Different variance/covariance structures will be explored (compound symmetry, first-order auto-regressive, Toeplitz and unstructured), and the structure that provides the best fit using the likelihood ratio test (or Akaike information criterion if compared models are not nested) will be used. Treatment comparisons will be presented as adjusted differences in means with 95% confidence intervals.

### Adjustment in models

The intention is to adjust all models for factors included in the cohort minimization: operation type (four different types) as a fixed effect and centre (17 different centres) as a random effect (or a shared frailty term in time-to-event models). Occasionally, the operation type might differ between the study database and the randomization system because it has been entered incorrectly into the randomization system. In this case, the value from the study database will be used, as the operation type recorded on the database will have been confirmed to be correct in such instances. For all treatment comparisons, the liberal group will be the reference group.

### Statistical significance

For hypothesis tests, two-tailed *P* values <0.05 are considered statistically significant. Likelihood ratio tests will be used in preference to Wald tests for hypothesis testing.

### Model assumptions

For all methods outlined, underlying assumptions will be checked using standard methods, for example, residual plots or log-log plots for proportional hazards. If assumptions are not valid, alternative methods of analysis will be sought (for example, by applying a logarithmic transformation or fitting a two-part mixed model for semi-continuous data [11]). If extreme outlying observations are found, whereby inclusion of such values results in an inadequate model fit, such observations will be excluded from the main analyses and sensitivity analyses may be performed to examine the effect on the study’s conclusions.

### Multiple testing

No formal adjustment will be made for multiple testing. However, the following measures to avoid problems with over-interpretation will be taken: (a) formal statistical comparisons will not be made for outcomes with low event rates, and (b) only pre-specified subgroup analyses will be performed and a significance level of 5% will be used for the tests for interaction for subgroup analyses despite being low powered tests. Consideration will be taken in the interpretation of results to reflect the number of statistical tests performed and the consistency, magnitude and direction of treatment estimates for different outcomes.

### Missing data

All missing data will be described by treatment group. If the amount of missing data differs substantially between groups, potential reasons will be explored. The following approach will be used to handle missing data in analysis models.

#### Missing predictor data

By design, there will be no missing data for any of the randomization factors. All other potential predictors are preoperative measurements of continuous longitudinal outcomes; by using the joint modelling approach described, missing values for such data are considered in the context of missing longitudinal data (see next).

Approaches for dealing with missing continuous outcome data measured at one time point are described in Table 4.

**Table 4:** **Missing continuous outcome data measured at one time point**

**Amount of missing data**	**Rule**
Less than 5%	Complete case analysis will be performed, that is excluding cases with missing data.
Between 5% and 15%	Marginal mean imputation will be performed, that is imputing the overall median or mean.
Between 15% and 25%	Conditional mean imputation methods will be used. This involves predicting the outcome from a regression model from (linearly related) covariates.
Above 25%	Multiple imputation will be considered. A general imputation model that uses an iterative procedure to generate imputed values will be used to generate multiple complete data sets. The model of interest will be fitted to each of the complete data sets and effect estimates combined using Rubin’s rules.

#### Missing longitudinal continuous outcome data

Preoperative values will be modelled jointly with those measured postoperatively, as described, thereby allowing all cases with at least one observation to be included. If appropriate (the level of missingness is >20%) then any variables that are predictive of missingness will be identified, and if there is reason to suggest that an assumption of missing at random given these variables is reasonable (this is especially likely if the variable was measured pre-operatively) then such variables will be adjusted for in the models of interest. These models can be shown to provide unbiased estimates of the treatment effect; moreover, multiple imputation approaches would not be expected to recover any additional information [12].

#### Missing binary or categorical outcome data

No formal imputation techniques will be used. However, for the primary outcome measure, the following approach will be followed. The element expected to have the highest amount of missing data is wound infection (identified via asepsis scoring [13,14]) measured in hospital and at 3-months follow-up. If in-hospital asepsis scores are missing and the following are true, the patient will be assumed to have no wound infection: (a) no antibiotics for suspected wound infection were prescribed in hospital, (b) follow-up is complete and the patient reported no problems with the healing of the wound at follow-up. Once this has been implemented, if the level of missing data is greater than 5%, this is likely to be mainly due to missing follow-up data and therefore separate treatment estimates will be made for the primary outcome: (a) at hospital discharge, and (b) at any time.

### Subgroup analyses

Seven pre-specified subgroup analyses of the primary outcome are stated in the study protocol: (a) operation type (isolated coronary artery bypass graft versus other operation types), (b) age at operation (<75 years versus ≥75 years), (c) preoperative diagnosis of diabetes (none versus diet, oral medication or insulin controlled), (d) preoperative diagnosis of lung disease (none versus chronic pulmonary disease or asthma), (e) preoperative renal impairment (estimated glomerular filtration rate ≤60 ml/min versus >60 ml/min), (f) sex (men versus women), (g) preoperative ventricular function (good (>50%) versus moderate or poor (≤50%)).

Each subgroup analysis will be performed by adding a relevant interaction term to the primary outcome logistic regression model (for example, for sex, a sex*treatment interaction term will be added to the model) [15]. The hypothesis for all subgroup analyses is that there will be no interaction. Odds ratios and 95% confidence intervals within each subgroup will be given alongside *P* values from the results of tests for interactions. *P* values for treatment estimates within each subgroup will not be given, unless a statistically significant interaction is found at the 5% level.

### Sensitivity analyses

The following sensitivity analyses have been identified; these were not pre-specified in the study protocol:

- Examining treatment estimates for the primary outcome by site, ordering sites by rates of severe non-adherence with the transfusion protocol: the hypothesis is that the treatment effect should tend towards the null with increasing non-adherence.
- Assessing the effect of the timing of primary outcome following randomization on the primary outcome by excluding all events that occurred in the first 24 hours after randomization: the hypothesis is that events in the first 24 hours are unlikely to be due to a post-randomization transfusion.
- Assessing the effect of the transfusions before randomization on the primary outcome by excluding patients who were transfused prior to randomization.
- Assessing the effect of acute kidney injury: acute kidney injury is defined according to the Acute Kidney Injury Network criteria [16] as either: (a) an increase in serum creatinine concentration (≥26.5 μmol/l, or ≥150% change from baseline) over a period of less than 48 hours, (b) restricted urine output (<0.5 ml/(kg h)) for more than 6 hours or (c) the need for renal replacement therapy. Highest daily creatinine levels are recorded separately from clinical judgment of acute kidney injury, so the following sensitivity analyses are planned to re-analyze the primary outcome: (a) excluding patients identified with acute kidney injury who do not have an increase in serum creatinine concentration over a 48 hour period or less, according to the daily highest creatinine concentration values collected, (b) including patients who have not been identified as having acute kidney injury, but according to the daily highest creatinine concentration data have a rise in serum creatinine concentration that would meet the criteria (and who were not having haemofiltration or dialysis pre-operatively).
- Serious primary outcome events: the pre-planned interim analysis after half the study participants had been recruited showed that the majority of the primary outcome events are either sepsis or acute kidney injury, and therefore the primary outcome will be re-analyzed including only the more ‘serious’ events. This will mean the following changes to the definition of the overall primary outcome: (a) all myocardial infarctions, gut infarctions and strokes will be included, (b) only the most severe acute kidney injury cases (stage 3) will be included, (c) all wound infections identified via asepsis scoring will be excluded (the more serious wound infections will be identified via serious sepsis events), (d) serious pre-discharge sepsis events will be identified by the presence of sepsis plus organ failure (defined as: myocardial infarction, stroke, acute kidney injury, laparotomy for gut infarction and one or more of reintubation, acute respiratory distress syndrome, low cardiac output or tracheostomy), (e) post-discharge sepsis events will be included, as they require hospitalization.

### Safety data

Adverse events will be tabulated by allocated treatment group; no formal comparisons will be made. Adverse events that meet the serious criteria (that is they (a) resulted in death, (b) were life threatening, (c) resulted in persistent or significant disability or incapacity, (d) prolonged an ongoing hospitalization or (e) resulted in hospitalization) will be identified (as serious adverse events) and all events will be divided into ‘expected’ events listed in the study protocol and other ‘unexpected’ events. Unexpected events will be independently coded by at least two trained research nurses blinded to treatment allocation using the Medical Dictionary for Regulatory Activities [17]. Any discrepancies between nurses in classification will be resolved by a cardiac surgeon also blinded to treatment allocation. System organ class terms will be used to group events, with groupings further broken down into preferred terms if necessary.

### Meta-analysis

It is intended to perform a meta-analysis combining the primary outcome results from this study with any previous systematic reviews and studies. This analysis will be performed using standard meta-analysis methods for binary outcomes, using a random effects model. Previous studies will be included in the meta-analysis if they fulfil the following criteria: (a) the patient population was patients undergoing cardiac surgery, (b) restrictive and liberal RBC transfusion strategies are compared, although the actual haemoglobin concentration and haematocrit thresholds for transfusion may differ between studies, (c) the outcomes included in the meta-analysis are postoperative morbidity or mortality.

### Pre-specified ancillary analyses

There are three pre-specified ancillary observational analyses in the study protocol:

1. Estimating the relationship between the number of RBC units transfused, and the risk of the primary outcome or death from any cause, stratified by trial arm.
2. Investigating the relationship between percentage decline in haemoglobin concentration from the preoperative level and the risk of primary outcome or death from any cause, taking into account the number of RBC units transfused.
3. Investigating whether the age of the RBCs is associated with the risk of primary outcome or death from any cause.

To address analyses (a) and (b), three logistic regression models will be fitted with the following explanatory variables:

1. Total number of RBC units transfused (either pre- or post-randomization)
2. Percentage decline in haemoglobin concentration from the preoperative level
3. Total number of RBC units transfused and percentage decline in haemoglobin concentration.

To address analysis (c), the age of the ‘oldest’ unit of RBCs received by a patient will be fitted as an explanatory variable. The age will be determined by linking the donation numbers of all RBCs transfused to a blood bank database and retrieving the date of donation.

In all of these models, adjustment will be performed for any variables found to be potential confounders, defined as: variables associated with both the exposure and the outcome that are not an intermediary step on the causal pathway between the exposure and outcome, that significantly contribute to the relevant multivariable model (defined as a likelihood ratio *P* value <0.05 or by modifying the effect estimate by greater than 10%). In analyses (a) and (b), the following variables have been identified as possible confounders: randomized allocation, operation type, centre (as a random effect), EuroSCORE, age and sex. Likewise for analysis (c), number of RBC units transfused, blood group, EuroSCORE, age and sex have been identified. For analyses (a) and (b), the instrumental variable method of controlling for confounding will also be explored, using randomized allocation as the instrumental variable [18].

Some potential issues have been identified. It may be sensible to restrict the analyses to include only patients who did not receive a proportionately large number of RBC units (for example, restrict the analysis to include those who received ten units or fewer). This approach would be used if, for example, the data obtained from patients who received large numbers of RBC units resulted in outliers and caused models not to fit adequately.

In all of the analyses (with the exception of decline in haemoglobin concentration) there is a potential problem that some of the RBCs might be transfused after a primary outcome event. Therefore fitting these models might not be appropriate, owing to the timing of the exposure relative to the outcome event. If this proves to be the case (for example, a non-negligible number of RBC units are given after the primary outcome measure or effect estimates do not make sense), alternative approaches will be considered [19], including:

- Nested matched case-control study: each patient experiencing a primary outcome event (‘cases’) will be matched to a ‘control’ (by matching on at least centre and randomized allocation); other factors (for example, operation type) may also be used if sufficient matched controls are available). For both the ‘case’ and the ‘control’, any RBC units transfused after the time that the case first experienced the primary outcome will be excluded from analyses.
- Time-to-event analyses with a time varying covariate of RBC units given: this would address the issue of exposure time (for ‘cases’, the event would be the primary outcome event, and for controls the last follow-up), but would ignore any blood given after the occurrence of a primary outcome event (that is, RBC units will only be excluded for ‘cases’).

For analysis (c), defining the age of the blood as the age of the oldest unit of blood transfused is likely to be confounded by the number of RBC units transfused. Therefore, the sensitivity of the results of this analysis will be explored by refitting the model using other definitions of the exposure variable, possibly including: the mean age of all RBC units, the use of any blood more than 14 days old (yes or no), the number or percentage of RBC units given that are more than 14 days old, the use of blood that is older than the median age of all RBC units transfused (yes or no). There are also potential problems with all of these approaches, for example, the use of any blood more than 14 days old is likely to be confounded by blood group and many of the methods that dichotomize patients into older versus younger blood will either need to exclude patients not transfused any RBC units, or to fit as a three-level variable of older blood, younger blood or no blood, which may in turn cause problems with interpretability.

### Changes to the original analysis plan

At the time of registering the trial protocol, a basic analysis plan was written. This has been followed when writing the current detailed plan, with some additions made, namely: details of variables to adjust for in analyses, rules for dealing with missing data, sensitivity analyses, meta-analysis and details of how the ancillary analyses will be performed.

## Discussion

We prospectively present the approach that will be taken in the analysis of the TITRe2 randomized controlled trial. Publishing the statistical analysis plan will increase transparency and promote deeper understanding of the methods used within the study. This transparency should reduce the risk of reporting data- or method-driven results.

During the peer review of this paper, it was pointed out that the marginal mean imputation method is not advisable for any level of missing data because it is likely to underestimate the variance of the treatment effect. We have not revised Table 4 because the analysis plan has since been executed and, in the event, this method of imputation was not implemented. However, we acknowledge that the method is inappropriate. We have revised the analysis plan template in our trials unit so that this method will not be proposed in further statistical analysis plans.

In preparing the statistical analysis plan, we have very deliberately sought to include our plans for additional, or secondary, analyses using trial data that are not directly related to the trial objectives. Doing this has compelled us to plan and consider the analysis approach and implications for the whole study collectively rather than in a fragmented manner. We believe that this has helped us formulate these plans more precisely and allows us to document that the plans were set out in advance of any data exploration. We recommend this approach to other researchers; otherwise, because the researchers are the ones performing the primary analyses, it can be difficult for them to substantiate a claim of pre-specification for a secondary analysis and to avoid criticisms of selective reporting in ways that have recently been identified in reports of the primary results of randomized controlled trials [20].

## Abbreviations

CONSORT: Consolidated standards of reporting trials
RBC: Red blood cell
